# Designing a Mobile App to Enhance Parenting Skills of Latinx Parents: A Community-Based Participatory Approach

**DOI:** 10.2196/12618

**Published:** 2020-01-24

**Authors:** Jennifer L Doty, Sonya S Brady, Javiera Monardez Popelka, Laura Rietveld, Diego Garcia-Huidobro, Matthew J Doty, Roxana Linares, Maria Veronica Svetaz, Michele L Allen

**Affiliations:** 1 Department of Family, Youth and Community Sciences University of Florida Gainesville, FL United States; 2 Division of Epidemiology and Community Health School of Public Health University of Minnesota Minneapolis, MN United States; 3 Hennepin Healthcare System Minneapolis, MN United States; 4 Department of Computer Science and Engineering University of Minnesota Minneapolis, MN United States; 5 Department of Family Medicine School of Medicine Pontificia Universidad Catolica de Chile Santiago Chile; 6 Best Buy Richfield, MN United States; 7 Centro Tyrone Guzman Minneapolis, MN United States; 8 Aquí Para Ti/Here for You Clinic for Latino Youth Hennepin Healthcare System Minneapolis, MN United States; 9 Department of Family Medicine and Community Health Medical School University of Minnesota Minneapolis, MN United States

**Keywords:** mobile application, eHealth, community-based participatory research, Hispanic Americans, family

## Abstract

**Background:**

Latinx families are among the highest users of smartphones, yet few health-focused Web programs have been developed for this audience. Parent-based smartphone apps designed for Latinx families may help increase access to evidence-informed parenting programming and ultimately reduce health disparities among children and adolescents. To maximize uptake of such apps, the Center for eHealth Research and Disease Management (CeHRes) Roadmap for electronic health (eHealth) development recommends 5 phases of development: (1) contextual inquiry, (2) value specification, (3) design, (4) operationalization, and (5) evaluation.

**Objective:**

Guided by the CeHRes Roadmap, our objective was to apply a community-based participatory research (CBPR) approach to mobile app development. We present a formative evaluation to inform the design of an eHealth mobile app for Latinx parents of adolescents based on a face-to-face parenting program, Padres Informados/Jovenes Preparados (PIJP).

**Methods:**

Community participants in the process included Latinx parents and stakeholders. We conducted a parent survey (N=115) and interviews (N=20) to understand the context and obtain feedback on a mockup and prototype of the app, facilitator workshops to streamline content, and stakeholder interviews (N=4) to discuss values and app requirements.

**Results:**

We report results from the first 3 phases of the CeHRes Roadmap. In the survey, 96.5% (111/115) of parents reported they had access to a cell phone, 85.6% (89/104) reported they would use a parenting app in the next month if they had access, and 80.2% (89/111) reported intentions to use a stress reduction app. Parents reported that setting goals about parenting and tracking those goals were important potential features of an app. In logistic regression analyses, technology attitudes and barriers were not related to parent’s intentions to use a parenting mobile app (95% CI 0.51-1.17 and 95% CI 0.28-2.12, respectively). Qualitative interviews confirmed Latinx parents’ technology engagement and desire for education and child development information online. Stakeholder interviews identified 3 community values: familism, the promotion of adolescent health, and delivery of economic value. Community stakeholders participated in defining the mobile app requirements. On the basis of community and parent input, the mobile app prototype was designed with 3 sections: (1) 8 modules of video-based parenting skills instruction with content from the face-to-face PIJP program, (2) breath rate information from a wearable device to support awareness of stress levels that could affect parenting, and (3) goal setting and tracking capacities.

**Conclusions:**

The findings of this study highlight the utility of an iterative, participatory design process. The CBPR approach and community collaboration enhanced the CeHRes Roadmap by promoting power sharing, facilitating recruitment, and building trust among community members. Experiences applying community research to the initial 3 phases of the CeHRes Roadmap in a Latinx community are discussed, along with plans for the 2 final phases.

## Introduction

### Overview

Evidence-informed parenting programs have been shown to improve parenting skills and reduce youth internalizing behaviors and substance use [1,2]. However, the reach of these programs is limited, particularly among Latinx families [1]. Mobile technology has the potential to aid in the dissemination of evidence-based parenting programs [3]. As Latinx families are high users of mobile phones, more specifically smartphones, parent-focused apps designed for this population may help increase access to evidence-based programming and ultimately reduce adolescent mental health and substance use health disparities [4,5].

### Background

Latinx individuals are leading users of smartphones [6]. From 2009 to 2016, the use of the internet in the United States nearly doubled among Spanish-dominant Latinxs (from 36% to 74%). In comparison, the use of the internet rose from 87% to 95% among English-dominant Latinxs and rose from 80% to 89% among whites. This narrowing of the gap in internet access is primarily because of the increased use of smartphones [6,7]. Broadband access to the internet among Spanish-dominant Latinxs was still relatively low in 2016 (ie, 21%) compared with 65% of English-dominant Latinxs and 73% of whites. These trends have prompted a call for health promotion efforts to focus on reaching immigrant Latinxs and Spanish speakers via mobile technology, especially through smartphones [5,8]. Despite the promise of mobile technology programs, 1 review of electronic health (eHealth) efforts found that less than 1% of programs were designed for Latinx communities [5], underscoring the need for research in this emerging area.

Uptake of mobile health technology is a critical concern to the success of eHealth interventions [9], and barriers to its successful uptake include low formal education levels, lack of health care access, and attitudes about technology or health, particularly for low-literacy and Spanish-speaking Latinx community members [8]. Community-based participatory research (CBPR) approaches may bridge trust gaps between the health care providers and community while marshaling support for eHealth implementation. Several key principles characterize CBPR approaches [10,11]. First, CBPR recognizes community as an entity that is a full partner at all stages of the research process [11,12]. Second, CBPR emphasizes egalitarian partnerships. Third, participatory approaches invest in change with the goal of reducing health disparities. Fourth, CBPR focuses on local problems, using a strengths-based approach. Fifth, cyclical processes are expected in CBPR research. Sixth, collaborating with different partners fosters humility, colearning, and capacity building. Seventh, CBPR includes commitment to a long-term investment with an eye toward sustainability. By involving end users throughout the development process, CBPR provides important strategies to build community perspectives and practical priorities into the design process, thus maximizing utility for Latinx parents [10,13]. CBPR increases translation of health equity programs into practice through participation of end users and stakeholders in the development and testing of interventions to improve their relevance, acceptability, and efficacy [14]. The CBPR approach recognizes the knowledge, expertise, and resources of communities.

### This Paper

In this paper, we apply a CBPR approach to the Center for eHealth Research and Disease Management (CeHRes) Roadmap for designing eHealth technology [13]. In particular, we illustrate the design of a smartphone app for parents, with the long-term goal of reducing internalizing symptoms and substance use among Latinx youth. We present the process of designing the PIJP mobile app, guided by the CeHRes Roadmap. The CeHRes Roadmap is a framework based on 16 models of eHealth development, incorporating an iterative process of design by integrating feedback from stakeholders and end users [13]. This makes it a human-centered process rather than a technology-centered process. Feedback should be recorded, and flexibly should be incorporated into the design of the app at every step of the process [15]. The CeHRes Roadmap outlines 5 phases of design for eHealth development [13]—contextual inquiry, value specification, design, operationalization, and evaluation (see Table 1). This has been described as a *participatory development approach* [13].

**Table 1:** Phases of Center for eHealth Research and Disease Management Roadmap: Application of community-based participatory research to Padres Informados/Jovenes Preparados app development.

CeHRes^a^ Roadmap phase of design	Phase description	CBPR^b^ application: PIJP^c^ app development	Progress
1. Contextual inquiry	Exploration of needs and strengths of users and community [13] Inclusion of multiple community viewpoints	We began with a commitment to understanding social, structural, and economic factors of the community [16]. A CBPR partnership comprised of representatives of the Latinx-serving community and researchers identified the need to have an app for parents [11,17]. A survey (N=115) and subsets of contextual interviews (n=20) were conducted to understand parents’ use of technology and needs and desires.	Complete (1-12 months)
2. Value specification	Documentation of stakeholders and potential users’ social and economic values [18] Translation of values into design and implementation considerations [13]	Via stakeholder interviews (N=4) and results of parent surveys and interviews, we identified familism and reducing adolescent health disparities as key social values. Increasing access to resources and keeping costs low were key economic values. Stakeholders agreed that an app provided a cost-effective means of disseminating the PIJP program. We chose a commercial rather than a research-focused wearable device because it was affordable.	Complete (3-12 months)
3. Design	Creation of a simple prototype or mockup of the proposed technology Iterative process of getting community feedback early and often on prototypes or mockups [13] Development of a business plan for disseminating the technology	We first created an interactive mockup using a tool called PopApp. The mockup was revised integrating results of the first set of contextual interviews with parents, and then, we created a prototype of the app. Community facilitators were cocreators in the translation of the content to short videos for the app [16]. In the second set of interviews, parents and community stakeholders gave feedback on the app prototype. We engaged in the initial business plan ideas to attain the CBPR goal of sustainability [10]. Ongoing discussions regarding this theme have continued.	In progress (6-18 months)
4. Operationalization	Implementation of the program, which may begin with pilot testing [13] Includes a plan for adoption in the community	Within the CBPR framework, community members, community organizations, and researchers will contribute to research design, recruitment, and evaluation [11,14]. Community members, including parents, will participate in an advisory board to successfully launch the app.	In planning (18-30 months)
5. Summative evaluation	Assessment of the impact of the technology on the community from a behavioral, organizational, and business perspective [8]	We will engage a broad coalition of stakeholders and community members to participate in evaluation efforts. Community priorities will be equally weighed with research priorities to make evaluation decisions [19].	Planned for future (30+ months)

^a^CeHRes: Center for eHealth Research and Disease Management.

^b^CBPR: community-based participatory research.

^c^PIJP: Padres Informados/Jovenes Preparados.

The purpose of this paper was to describe how a community-based participatory approach was applied to the adaptation of the face-to-face PIJP curriculum and the development of the mobile app. We had the following aims: (1) to explore Latinx parents’ access to technology, current use of technology, and intentions to use a parenting app, based on a mixed methods contextual inquiry; (2) to identify the stakeholder values and parent desires for a parenting/stress reduction app and wearable device to support awareness of stress levels that could affect parenting; and (3) to synthesize and integrate parent feedback about the mobile app mockup and the working prototype into the design of the mobile app.

In this paper, we discuss the findings, as they are relevant to the 5 phases of the CeHRes Roadmap. As a method of conducting research that is rooted deeply in community involvement, CBPR naturally fits with the participatory design process of CeHRes.

## Methods

### The Padres Informados/Jovenes Preparados Program

The Padres Informados/Jovenes Preparados (PIJP) program was developed using a CBPR approach. The PIJP partnership began in 2007 to support parents and youth to reduce smoking by developing a culturally-grounded parenting program. The development of PIJP included Latinx-serving organizations and Latinx parents of youth who helped align parent and community priorities. Guided by social cognitive theory and positive youth development principles [20,21], PIJP focuses on core cultural values while building family relationship characteristics and parenting practices most closely associated with substance use prevention. Parenting practices include monitoring of children’s whereabouts and activities as well as the use of effective and consistent discipline. As part of the face-to-face delivery of the PIJP program, parents attended 8 weekly 2-hour sessions. A total of 4 sessions of a complementary youth program were delivered conjointly with the parent sessions. The program and face-to-face delivery are described in greater detail in a study by Allen et al [22]. Results from a randomized, wait-list trial (2011 to 2013) demonstrated that the program lowered intention to smoke among youth who reported low levels of traditional Latinx cultural values. Family relational training resulted in improved family relations characterized by increased attachment, warmth, and support; decreased conflict; and increased acceptance and constructive communication [23].

Building the PIJP mobile app was part of a larger action-oriented dissemination plan—a call to action in response to community needs [11,17]. Dissemination of the PIJP program occurred through a 3-pronged approach: (1) developing a website to easily disseminate the program materials to local facilitators, (2) piloting a *train-the-trainer* model to ensure fidelity of delivery, and (3) developing a mobile app that parents could access directly. A website was developed to disseminate PIJP program materials, and the program has been disseminated using the train-the-trainer model in a metropolitan area in the Midwest of the United States. Methods to develop the mobile app are described in the following sections.

### Community Stakeholders and Research Team

A multidisciplinary team of PIJP stakeholders—critical for eHealth research and development [13]—has continued to regularly attend a monthly community-participatory research meeting for over 10 years. As the dissemination phase launched 5 years ago, 2 authors (JD and JM) joined the collaborative and have led the mobile app development. The PIJP stakeholders include leadership from a local community serving agency, a physician from a Latinx-serving clinic, a public health outreach specialist, and university researchers and extension specialists. To lay the groundwork for the PIJP app, stakeholders discussed and approved a mixed methods research plan to survey local Latinx parents, interview a subset of parents about their technology use, and obtain feedback on the app design.

A graduate student with experience in human-computer interaction and prototyping, an undergraduate intern, and JD met with a user experience and design expert with over 15 years of experience in the industry to formulate questions and discuss how to translate requirements into design. In addition, 8 stakeholders were invited to participate in individual interviews, and 4 stakeholders participated in 1-hour interviews outside of monthly meetings to gather requirements. The graduate student interviewed stakeholders, while the undergraduate intern took notes. Stakeholders’ and parents’ values were translated into design requirements, keeping communication open throughout the design process so as not to isolate or lose ideas [15]. Experienced Latinx community facilitators of the PIJP program became cocreators of the content that would be selected for the mobile app version of the program. These facilitators were previously trained and had presented the face-to-face PIJP program in the community [19].

### Research Design

**Figure 1:**
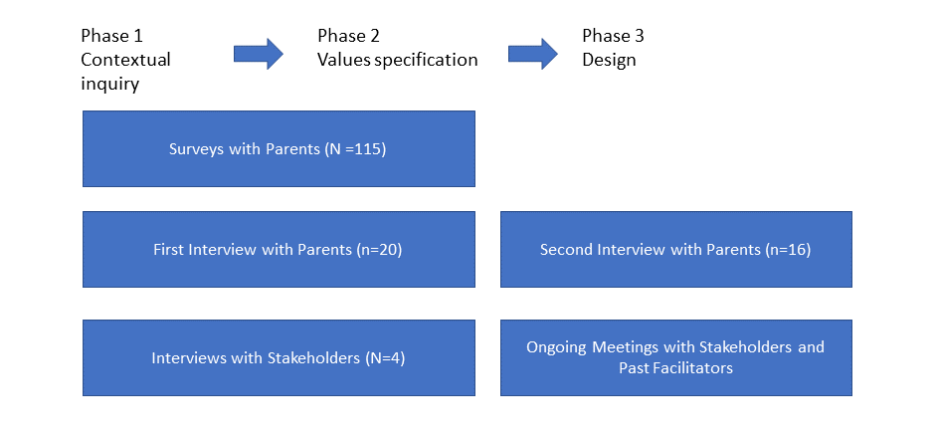
Summary of study design.

We employed a mixed methods design for the formative evaluation to triangulate data and better understand parents’ needs (see Figure 1) [13]. We conducted a quantitative survey (N=115) to achieve a big-picture overview of Latinx parents’ technology use. We then conducted 2 sets of qualitative interviews to gather parent feedback on the usability of the design [24]. The first interview (approximately 60 min; n=20) covered parents’ online interactions, use of technology for parenting, stress reduction resources on the Web, and feedback on the mockup of the mobile app. Our mockup was created using Pop, an app by Marvel, which allowed a simple but interactive prototype of the app using PowerPoint images.

Design of the prototype of the app began 4 months later. Applying Persuasive Systems Design (see Multimedia Appendix 1), we focused on aspects of the app design intended to increase primary task support (eg, increase the target behaviors of positive parenting), dialog support (eg, increase feedback the app gives to parents), and system credibility (eg, increase trustworthiness and real-world feel) [25]. A second interview (approximately 45 min; n=16) was conducted to gather feedback on using the mobile app prototype and parents’ experience using a wearable device. We invited parents to *think out loud* about the process of navigating through the app. This method has been recognized as a tool for gathering participant feedback in the development of eHealth technologies [26] and fits with the CBPR commitment to incorporate opportunities for community members to provide meaningful input [10,16].

### Participants

#### Surveys

We recruited Latinx parents (N=115) through flyers, word of mouth, tabling at community events, and face-to-face invitations to participants in classes at local agencies. Most respondents either approached our table at 3 large community events where more than 100 community members were in attendance or participated after being invited in community classes with 12 to 18 participants. Recruitment was stratified by gender with the goal of recruiting 40% of fathers. All surveys were given in Spanish, with the option to take them in English, and participants had the option to complete the surveys on paper or a tablet.

#### Interviews

Of the 70 survey participants who indicated in the survey that they would participate in interviews, 30 were randomly chosen and invited to meet with the outreach specialist. The first participants who were able to schedule an appointment were interviewed for both the first (n=20) and second phases (n=16) of qualitative interviews. The outreach specialist met participants for interviews at the location of participants’ choice (eg, home or local cafe) to conduct 2 interviews in Spanish. Interviews were recorded, transcribed, and translated into English using an online service, and interactions with the mockup and prototype were recorded using a portable IPEVO camera.

### Measures

#### Quantitative Measures

Stakeholder input was incorporated into survey development, and parents gave feedback on a draft of the survey. Access to mobile technology was measured by the following questions: “Do you access the internet on a cellphone?” (1=yes and 0=no) and “Do you have a data plan for your phone?” (1=yes and 0=no). Intention to use a parenting app was measured by the following question: “If a parenting/biofeedback app were available today, I predict I would use the mobile app in the next...” (1=week to 7=never). Parents indicated the importance of parenting information in response to the following question: “Which of the following things would be most important to you in a parenting app?” Responses included “Information about communication or connecting with your child”; “Information about negotiating culture and adolescent development”; “Information about discipline, monitoring my child, conflict resolution”; and “Connections to other parents.” Parents indicated the importance of potential design features in response to the following question: “Which of the following things would be most important to you in a parenting app?” Responses included “Stress reduction and relaxation tips,” “Personal feedback on my stress levels through a wearable,” “The ability to write and track goals,” and “Reminders about my goals on my phone.”

Technology attitudes were measured using a 6-item scale (alpha=.62) [27]; for example, “Thinking about all the technologies you use, overall would you say these devices: ‘Make your life easier’ or ‘Give you less control over your life’” (1=*strongly disagree* to 5=*strongly agree*). To measure perceived barriers to technology use, parents answered the question, “Which (if any) of these things make it difficult for you to use or concern you about the Internet?” [27]. A count variable was created from a checklist of 7 items, such as “I’m worried about computer viruses.” Control variables included age of parents, gender (0=female and 1=male), educational attainment (1=did not go to school to 5=university), and monthly income (1=less than US $1000 per month to 5=more than US $4000 per month).

#### Qualitative Protocol

The first parent interview covered 4 topics: general technology use, use of technology for parenting, preferences for the app, and feedback on the mockup of the app. Interview questions are found in Multimedia Appendix 2. The first parent interview was informed by parents’ responses to the quantitative survey*.* For example, parents emphasized making parenting goals and were enthusiastic about using an app to support parenting and stress reduction. We asked further details about these topics in the interviews. In the second parent interview, parents reported on their preferences for 8 potential features that could be included in a parenting app. To facilitate the process of determining preferences, parents were presented features in 7 sets of 4 and instructed the following: “Think about the features in each example set of an app. Which would be the most important and least important characteristics to you?” Some example options include the following: “The app is generic—it does not display personalized information about you or your child” or “You are able to make parenting goals in the app based on the parenting module and track each day of the week.” Parents indicated the most and least important features in each set. Guided by an expert in the user experience field, we took a practical approach to investigating user experience. Specifically, we conducted user testing, and participants talked out loud about their experience completing tasks in the app prototype. For example, we asked them about the first page, and before clicking on anything, participants shared their first impressions of who the app was for and the purpose of the app. Then, participants were given a series of scenarios and asked to navigate through the prototype, for example, “If you had a hard day at work and wanted to relax for two minutes before helping your child do homework, what part of the app might be helpful?” In the stakeholder interviews (N=4), we asked partners about the future of the PIJP program and collaborative, their understanding of the project to build a mobile app, the benefits of the project to the community collaborative, desired features and functionality of the app, potential problems with the app, and successful implementation of the app.

### Analytic Plan

Descriptive statistics were computed on the survey data to understand parents’ access to and current use of technology as well as their intentions to use a parenting app. Logistic regression analyses were conducted to assess the relationships of technology attitudes and barriers with access and use patterns. To address missing data, we conducted 25 iterations of multiple imputation in Stata.

For qualitative analyses of parent and stakeholder interviews, the first set of interviews were coded by JD and the outreach specialist to reduce the bias of 1 researcher interpreting the data [28]. We met on 2 occasions to discuss coding and reach consensus regarding themes that emerged and design implications.

## Results

Survey participants (N=115) included 59.8% (64/107) of mothers. Nearly three-fourths (78/107, 72.9%) of the participants were married, and the mean age was slightly over 40 years (mean 40.6). Approximately half of the participants reported technical training or education beyond high school (56/110, 50.9%), 77.4% (89/115) were from Mexico or Central America, and 18.5% (20/108) reported being in the United States for less than 10 years. A subset of 20 survey participants were interviewed on 2 occasions. Of this subset of participants, nearly three-fourths were mothers, 68% (13/19) were married, and the mean age was slightly over 41 years (mean 41.5). Slightly more than half of the participants reported technical training or education beyond high school (12/20, 60%), 65% (13/20) were from Mexico or Central America, and 20% (4/20) reported being in the United States for less than 10 years.

### Context and Values

The CBPR approach supported both quantitative data collection and 2 rounds of qualitative data collection necessary for the iterative process of design (see Table 1). In the first 2 phases of the CeHRes Roadmap, we focused on contextual inquiry and value specification.

#### Quantitative Survey

We found that 96.5% (111/115) of parents had access to a cell phone, whereas only 68.8% (77/112) had access to a home computer. Parents reported that 84.5% (93/110) had access to a data plan. Parents also indicated that they would use a parenting app within a month if they had access to it (89/104, 85.6%), and 80.2% (89/111) indicated that they would use a stress reduction app within a month. We conducted a logistic regression analysis to examine the relationship between technology attitudes with intentions to use a parenting app in the next month. We found no relationship (odds ratio [OR] 0.77, 95% CI 0.51-1.17). In addition, technology barriers were not related to intentions to use a parenting app (OR 0.76, 95% CI 0.28-2.12).

#### First Set of Parent Interviews

Some of the Latinx parents we interviewed were avid technology users, whereas some were weary of technology. However, all parents we interviewed said they used basic apps including email, WhatsApp, Google, Facebook, and banking apps. Some parents listed more than a dozen apps that they used on a regular basis to research hobbies, connect with their child’s school, travel, engage in entertainment, or transfer money. Few barriers to technology were mentioned. Moreover, 1 parent mentioned a lack of access, and another mentioned privacy concerns. Some parents expressed frustration that technology is always changing, and they depended on their children to help them learn to use new devices and software. One parent said:

What I find difficult is when I don't use, for example an Xbox...I usually learn how to use whatever I have. And when something is very difficult, that I don't understand, I call [my children], and they make everything easier for me.

Few parents had used wearable devices or relaxation apps. A couple who had tried relaxation or mindfulness apps for a short time did not continue using the app. For example, one said:

I deleted it, I got bored, I used to fall asleep.

Other methods of relaxing using technology were common. One mother said:

[To relax, I use] my phone to make a call, and sometimes watch a video. I look for healthy food and exercises on Google...I like to see how to feed my family and how to feed myself, what exercises are good and how to do them.

Others avoided technology, and another mother said:

When I’m stressed I prefer to get away from [technology]…For example, I read a book and stop working on the computer.

When it came to parenting, parents saw value in technology as a parenting and education resource. Almost all parents communicated with their child’s school and monitored their academic progress on the Web. Overall, they were enthusiastic about the role of technology in their children’s education. A parent explained:

I also opened an email account for communicating with the teachers...Now with the internet, I realized that it is a big help for school because, for example, my children are learning now about math, and I remember it was more difficult for me to learn math. Instead they are like playing, counting, so math is easier.

They saw technology as a potential way for them to learn information about parenting too. One parent said:

When I want to learn things, what I do is Google them; my daughter is a teenager, and there I start reading a bit more about how to handle some situations that they start having during adolescence.

Parents also wanted information on parenting by developmental ages:

I think there are so many resources available on internet, but sometimes it’s difficult to find them, so, I’d like a place where it only focuses on parenting, education, or children, divided by ages.

Another parent said:

I would like [an app] to talk about how to help students, how to be parents, how to help each other.

A few acknowledged the need for support as they navigate a new culture:

[I’d like] something that helps us to understand a little more about the culture, to help us adapt a little more to the new culture.

Some wished that they had a way to monitor their children’s use of technology. A couple said that they thought adolescents needed independence from monitoring. Overall, parents were positive about the potential for technology to support their family and parenting efforts.

#### Stakeholder Interviews

When stakeholders were asked the direction in which they would like the PIJP program to progress over the next few years, stakeholders articulated a goal of helping parents gain parenting skills by having access to the material they need when they need it. Stakeholders mentioned the desire to provide access and flexibility but also mentioned the need for sensitivity to family data limits. Regarding the features and functionality of the app, all 4 stakeholder participants were enthusiastic about the possibility of including breath rate feedback on stress levels to parents via a wearable device and reminders to parents through the app. One stakeholder commented, although, that “different people will have different reactions to notifications” and noted that we would need to study this further. Stakeholders also agreed on the need to have interactive audio/visual and brief time windows to present material. All stakeholders suggested promoting family connections and social support through the app, perhaps by using social media. Others suggested a hybrid program with personalized training and consultation provided alongside the app. Each stakeholder we talked to was interested in developing more specialized content on youth issues (eg, information on nutrition, sexual health, and mental health). When we asked how we could successfully implement the dissemination of the app, stakeholders mentioned “grassroots growth,” recognition from local health systems, and the need for “investment from funders.” Other ideas to incorporate into a business plan included conducting an initial in-person provider and parent meeting, planning for ongoing improvements to the app, leveraging the app to provide referrals to caregivers, and incorporating video conferencing into the app over time. Stakeholders not only recognized the potential value of the app in clinical practice (eg, at doctors’ visits or in meetings with social workers) but also thought that “we’re a long way from using this in routine clinical practice” because mobile dissemination would need to be systematically tested. Sustainability of the app economically and long-term commitment to the project and community were also valued.

### Design

#### Stakeholder Interviews

The results of the stakeholder interviews were presented to all stakeholders (including those who did not participate in interviews) at a monthly meeting in the form of updated requirements and a proposed site map (see Multimedia Appendix 3). On the basis of stakeholder interviews, the team agreed that future releases of the app should include additional content on specialized areas of youth development and exploration of social media connections. In addition, stakeholders discussed the potential future release of a youth version of the app. Stakeholders agreed with this plan, given the limited budget for the first release. Most points of disagreement involved presentation of content (eg, the extent to which we could include gender-neutral language). Another area of some disagreement and discussion was the need for a wearable device that would be affordable for participants, rather than a costly research-grade wearable device. Some stakeholders argued that affordability was critical to sustainability of the project over time. Disagreements were resolved over several months after having discussions in monthly meetings with stakeholders and gathering opinions from community members. CBPR values of colearning, humility, and shared power were critical to resolving differences.

On the basis of stakeholder feedback, the app prototype featured a homepage that directed parents to 1 of the 3 areas: (1) parenting tools with 8 modules on parenting practices; (2) relaxation techniques, which would connect to breath rate information from a wearable device and breathing exercise apps; and (3) goals, which provided an opportunity for parents to make and track goals about parenting and relaxation practices. As stakeholders were concerned about parents not being able to stream if they did not have data, we included transcripts of videos.

#### Second Parent Interview: Feedback on Design

The same parents who participated in a user test of the mockup of the Padres Informados app in interview 1 were invited to provide feedback on the prototype of the Padres Informados app in interview 2 (n=16). Parents were confused by some of the labels, but most were able to complete each task (finding information on discipline, relaxation, monitoring their breath rate, and setting a goal). The only task that sometimes gave parents problems was setting up their personal preferences. During the interview, parents were asked to choose the most important and least important features of the app from a menu of 8 options presented in sets of 4. Parents indicated that the most important features of the app were setting goals (40%) and tracking on a weekly or monthly basis (40%). Parents indicated that the least important features of the app were lengthy videos (40%) and prompts to finish all 8 modules (20%).

## Discussion

### Principal Findings

This study supports the utility of combining the CeHRes Roadmap with CBPR methods for designing eHealth technology for Latinx families. Using mixed methods, the contextual inquiry phase of the CeHRes Roadmap included a survey of Latinx parents and interviews with a subset of parents. The findings of this study highlighted parents’ engagement with technology and illustrated parents’ desires to improve their parenting skills and knowledge. Latinx parents in the local community were connected to technology. Similar to national findings [7], 96.5% of parents had cell phones, and the majority of parents indicated that they would use an app for parenting (85.6%) or stress reduction (80.2%) if it were available. Technology attitudes and barriers were not associated with parent’s intentions to use a parenting app or wearable device. Although 84.5% of parents reported having a plan for data usage, stakeholders who reviewed survey data were nonetheless concerned that some families would not be able to stream videos.

During qualitative interviews, parents indicated that one of the most important features of a parenting app would be goal setting and tracking capabilities. Interviews illustrated the ways in which Latinx parents wanted to improve their parenting by learning about the developmental stages their children were experiencing, how to discuss sensitive subjects, and ways to better monitor their adolescents. The interviews also demonstrated how Latinx parents used technology in their everyday lives; parents discussed several apps they use daily, but few parents had tried a wearable device or an app focused on relaxation. Interviews with stakeholders further identified the values of community stakeholders, which were translated into requirements for the development of the app.

In the following paragraphs, we discuss the benefits of a CBPR approach to the formative evaluation and application of results to the design of the PIJP app in the first 3 phases of the CeHRes Roadmap. We also discuss future directions for this effort in the last 2 phases, encompassing operationalization through piloting and the implementation and evaluation of the mobile app with community partners.

### Contextual Inquiry

The first phase of the CeHRes Roadmap, contextual inquiry, informed the initial planning of the app by soliciting multiple viewpoints and exploring the needs and strengths of the community. A contextual approach links to a key principle of CBPR, the commitment to understanding social, structural, and economic factors that may contribute to inequalities [16]. Working with community as full partners facilitated recruitment for the parent survey and interviews, which is a valuable contribution given the difficulty of engaging busy parents [29,30]. Other studies have demonstrated that commitment to include community stakeholders and members increases trust, in part, because potential end users of eHealth technology have familiarity with and input into product development [13].

Stakeholders had already identified the dissemination of the PIJP program as a high priority and agreed that building an app was an innovative and sustainable means to deliver the program to parents. In another PIJP study, researchers found that fathers were less likely to attend meetings face to face compared with mothers; program delivery via technology was identified as a means of increasing fathers’ participation [31]. Other research has also emphasized the importance of a CBPR approach for engaging stakeholders and community partners in eHealth development to improve the sustainability of product use over time [32]. In this study, surveys, the first set of parent interviews, and stakeholder interviews informed the expansion of user requirements with the following additional features: inclusion of goal tracking and reminders for goals. Guided by the parents’ feedback during the second set of parenting interviews, we decided to focus most on foundational parenting information and goals in the first release.

### Value Specification

During the second phase of the CeHRes Roadmap, 3 values were identified by stakeholders and parents: familism, adolescent health, and sustainability through economic value. Consistent with formative research conducted for the face-to-face version of PIJP, the value of familism was identified as a social value [22]. Familism refers to the strong sense of importance and connectedness in family relationships and obligations among Latinx people [33]. Parents reported that improving parenting knowledge and supporting their children’s education were key priorities, reflecting the value of familism. Furthermore, parents identified tracking of parenting goals as the most important features of a parenting app, as they responded to a ranking task about important features of the app.

Stakeholders also identified the value of improving adolescent health. They requested future modules on adolescent health topics, such as youth substance use, nutrition, and sexual health. On the basis of the stakeholder interviews, the site map of the proposed mobile app demonstrated the features of the app that we planned to design and showed the priority of each feature for each release. In the first release, we focused on basic parenting modules and goal setting capacity, and in subsequent releases, we plan to focus on wearable integration, social connection, and future topical modules on adolescent health. These decisions were also informed by the parent survey and interviews. In line with the CBPR principles of shared power, the monthly meeting to obtain stakeholder feedback and refine the site map facilitated the integration of community knowledge into the CeHRes Roadmap process [10,16].

The PIJP community-academic group also discussed the potential for economic value, with a focus on sustainability of the PIJP mission and outreach, rather than profit. Stakeholders highlighted the importance of community access as a key value, and we discussed this further in monthly meetings. The possibility of a no-cost or low-cost app was appealing, especially in comparison with some costly programs. When we first discussed integrating a wearable device into the app, cost was a major concern. Some research-intensive wearables have multiple monitors and a focus on measurement [34,35], but Latinx parents in the community were unlikely to buy these or wear them on a regular basis. Furthermore, research-intensive wearables did not have the user-friendly interface that would allow parents to monitor their own stress data. We decided to do feasibility testing with the Spire stone, a commercially available, user-friendly wearable device that tracks breath rate [36]. Our focus in choosing this product was on accessibility, ease of community use, and feasibility from users’ perspective [37]. However, although the wearable device complements the PIJP app, it is not necessary for parents to use the wearable device and engage in relaxation exercises to benefit from the app. The decision to create an accessible product rather than a research-intensive product and to make the wearable device an optional feature would not have been considered without the CBPR approach, which demonstrates that the insights of local stakeholders make valuable contributions to eHealth mobile app development.

### Design

In the CeHRes Roadmap, design is defined as “building prototypes that fit with the [identified] values and user requirements [13].” The CBPR process provides a structure to identify values and requirements that meet the needs of marginalized communities. Through close collaboration with the community, the PIJP team was uniquely positioned to design a mobile app based on human-centered design principles. These design principles include a minimalist aesthetic and design, clear navigation, structural consistency, and heavy dependence on understanding and being responsive to users’ needs and skills [38,39]. Inherent in the CeHRes Roadmap is the recognition of persuasive design as a key principle of eHealth development [13]. Working from the CBPR principle of leveraging community assets, we chose to apply a structured model of Persuasive Systems Design, which presents a process of designing eHealth products to “reinforce, change, or shape attitudes or behaviors or both without using coercion or deception” [25]. Collectively, these design principles supported a focus on creating a user-friendly app, identifying salient PIJP content, and making decisions that promoted the feasibility of parents using the app.

The CBPR process leveraged the deep knowledge and experience of Latinx community facilitators, stakeholders, and parents in shaping the delivery of content for the app. Community facilitators of the PIJP program became cocreators of content that would be selected for the mobile app version of the program. These facilitators were previously trained in and had presented the face-to-face PIJP program in the community [11,19]. Stakeholders and community facilitators of the face-to-face program recommended delivering the content through short videos based on their knowledge of the community. The use of videos for the delivery of parenting information in prevention settings has been previously validated [40]. In addition, videos have been recommended for delivering information to Latinx participants in eHealth contexts [5]. We then validated the idea of videos by asking parents to identify the most important and least important potential features of the parenting app. Although parents did not like the idea of long videos (25-30 min in length), they were open to the idea of short videos (3-5 min in length). To address parents’ potential inability to stream content, we also plan to include the written script to each video in Spanish. Parents’ and stakeholders’ feedback on the prototype of the app will be incorporated into the final product, including emphasis on goal setting, shorter videos, and some minor changes to labeling of different app sections. Consistent with a CBPR approach, we engaged stakeholders, facilitators, and parents to ensure community members were cocreators in this process [16]. When communities are invested in the process, acceptability of the end product is more likely [13].

### Operationalization

Dovetailing with the CBPR commitment to sustainability, the operationalization phase of the CeHRes Roadmap focuses on both pilot testing and business modeling. This dual process is important to establish viable and sustainable means of implementation, a critical process gap between the development of health care innovations and implementation in health care settings [41]. The operationalization phase and evaluation phase (below) represent future directions for the PIJP app. The pilot study will be conducted in close partnership with the Latinx community, and community members will be a part of the research process [11]. Simultaneously, the process of creating a business plan in a CBPR context requires negotiation and open discussions about partnership [10,11]. This process will help us identify how PIJP creates and delivers value. Furthermore, a business model will identify regulations, opinion leaders in the community, incentives that work with local parents, and opportunities to collaborate with insurance plans [13]. This process includes identifying key partners, resources, activities, and the cost associated with implementation as well as potential customers and distribution channels that may provide revenue. Having a business model before the implementation is critical to successful integration into health systems [18].

### Summative Evaluation

The tensions between evidence-based practice, community priorities, and business models need to be weighed carefully in the evaluation phase. For a summative or outcome-focused evaluation, the gold standard for evidence-based practice is a randomized controlled trial (RCT). However, although an RCT may meet research needs, this design lacks the flexibility to meet community needs for those who are in the control group [11]. Therefore, we plan to conduct an optimization trial before considering an RCT. In the context of this project, optimization refers to the process of identifying which components of the app and support systems (such as SMS reminders, weekly check-ins with community health workers, and training on the app) provide the best outcomes at a reasonable cost [42]. To establish the effectiveness of the mobile app and the impact on outcomes, after the design phase, summative evaluation is necessary [13]. To this end, we will conduct an RCT after the optimization trial. However, the idea that an evidence-based product should not change after positive results of an RCT is based on an assumption that the context of participants is not changing. Especially in a rapidly changing technological landscape, evidence-based practice should be coupled with a business model dedicated to ongoing evaluation and assessment of the impact of eHealth technologies on client behaviors, clinical practice, and health outcomes [13]. In the case of PIJP, ongoing collaboration with community stakeholders using CBPR supports long-term sustainability of a product and the commitment for the development and summative evaluation of future planned iterations of the product.

### Limitations

This study follows a carefully designed formative evaluation of community needs and adheres to a well-respected process of building eHealth designs, the CeHRes Roadmap [13]. However, limitations must be acknowledged. Not all stakeholders we contacted were available for interviews, and we instead gathered feedback from some stakeholders at monthly meetings. We used word of mouth and community agencies to recruit participants, resulting in a convenience sample. As a result, the sample may be positively biased toward technology compared with those who did not participate in the formative study. In addition, we were not able to statistically analyze parent’s rankings of best/worst choices of the app features after the interviews. To conduct a best/worst scaling analysis, future studies should be conducted with larger numbers of participants in a survey format [43]. Designing at the community level may meet the needs of local communities but may not be transferable to a greater audience; a balance between meeting community needs and being able to impact population-level health must be reached. It should be noted that the effectiveness of an app may be diminished if participants are unaware of its full functionality. We believe that CBPR is an approach that maximizes the effectiveness of programs, communication with participants, and adaptability of programs at the community level while adhering to evidence-based principles.

### Conclusions

The integration of CBPR in developing eHealth technology for the Latinx community has enabled a critical feedback loop of community input into the design process. More specifically, the CBPR approach supported recruitment of parents in the community; built trust through the endorsement of community stakeholders; and leveraged the knowledge of stakeholders, prevention program facilitators, and parents in the process of app development. The PIJP commitment to a CBPR approach added a critical analysis of cultural needs and addressed power sharing in design decisions and business plans, which has the potential to effectively promote health equity [10,16]. By applying CBPR to eHealth technology development and business modeling, technology can be built in such a way to have a wider benefit and expand access for community members. A CBPR framework has the potential to add value to the CeHRes Roadmap and the process of eHealth adaptation of other family-based interventions for Latinx families.

## Appendix

Multimedia Appendix 1 Persuasive Systems Design model applied to the Padres Informados/Jovenes Preparados app.

Multimedia Appendix 2 Example questions of qualitative interviews.

Multimedia Appendix 3 Proposed site map of the Padres Informados/Jovenes Preparados app.

## Abbreviations

CBPR: community-based participatory research
CeHRes: Center for eHealth Research and Disease Management
eHealth: electronic health
OR: odds ratio
PIJP: Padres Informados/Jovenes Preparados
RCT: randomized controlled trial
