# Supplementary material for: Designing a Mobile App to Enhance Parenting Skills of Latinx Parents: A Community-Based Participatory Approach
Source: JMIR Form Res. 2020 Jan 24;4(1):e12618. doi: 10.2196/12618 (PMC7007588; doi:10.2196/12618)
Supplement: Multimedia Appendix 1 [file formative_v4i1e12618_app1.docx]

Multimedia Appendix 1. Persuasive Systems Design Model Applied to the PIJP App

| **PSD Principle** | **Example requirement** | **Example implementation in the PIJP app** |
| --- | --- | --- |
| **Primary Support** | | |
| Reduction | Reduce parenting, a complex process, into simple tasks | - Through CBPR and a review of parenting literature, parenting skills (tasks) were identified. - Latinx parents prefer videos to written content. Each short video covers one parenting skill. |
| Tunneling | Provide an experience for parents that provides an actionable pathway toward the target behavior | - At the end of modules, specific goals are suggested for improving parenting skills; parents can also write their own goal. |
| Tailoring | Provide tailored content according to parents’ needs, wants, and contexts | - A brief parenting style assessment was added to the first module to help parents tailor their experience. - Parents can choose modules that best meet their parenting needs. |
| Personalization | Give parents personalized content and modules | - Parents can personalize the app by entering their child’s name and gender. - Parents will have a personalized dashboard. Module recommendations will be based on parenting style.^a^ |
| Self-monitoring | Provide feedback on progress and status on goals | - Parents can see the badges they have earned for completed modules and modules they have yet to complete. - The goals section has tracking available weekly. Past weeks are available for review. - Breath rate information will be available once the wearable is integrated.^a^ |
| Simulation | Include scenarios that model cause and effect in parenting behaviors | - Modules include videos of actors modeling parenting scenarios to illustrate cause and effect. |
| Rehearsal | Allow parents to rehearse parenting skills through interactive activities | - For each parenting skill, interactive practice activities will be included (e.g., journaling, choosing best scenarios).^a^ |
| **Dialogue Support** | | |
| Praise & Rewards | Provide positive reinforcement for completion of modules | - Parenting style strengths are highlighted. - Parents are praised at the end of each module and quiz with a badge. - Parents can see their collected badges. |
| Suggestion | Suggest specific behaviors for practicing the skills in each module | - At the end of each module parents are given goal setting options, suggestions for a behavioral goal from a drop-down menu, or the option to write their own goal. |
| Reminders | Prompt parents to work towards their goals | - Parents are sent notifications to remind them to track goals. |
| Similarity | Design meaningful features that resonate with parents | - At the outside of the intervention, culturally appropriate pictures and scenarios are approved by community facilitators and parent advisors. - Parents will identify their main motivation for completing the program. Motivation statements will be visible in the goal section of the app.^a^ |
| Liking | Design a visually attractive interface | - Bright colors and a clean design were prioritized. - Feedback from parents in user testing was integrated. |
| **System Credibility** | | |
| Trustworthiness | Ensure information is trustworthy and unbiased. | - Information was based on an evidence-informed program developed with community partners. |
| Expertise | Reflects knowledge, competence, and experience | - A brief explanation of the community based participatory research process was given in the introduction to what? with the option to link to the full website with research information. - App was kept up-to-date. |
| Surface credibility | Inspires confidence with a professional looking design | - Consultation with user experience professionals (Phase 1) and a designer (Phase 2) ensure a clean, professional look. |
| Real-world feel | Share information about the organizations behind the app. | - Each partner in the Padres Program is highlighted in the information links. |
| Authority | Leverage endorsements by authority figures | - In the context of CBPR, authority is replaced with earned respect from the community. - Recognition and endorsements of the website from respected community leaders was included. |
| Third-party endorsements | Highlight endorsements from well-known respected sources. | - Endorsements from parents who have completed program will be included. |
| Verifiability | Provide outside sources to verify the accuracy of the content. | - Publications will be available via an external link. |
| **Social Support^b^** | | |
| Social learning | Opportunities to observe other parents demonstrating positive parenting behaviors. | - Videos demonstrate scenarios where different parenting styles are used. |
| Social comparison | Provide means to compare with other parents. | - Parents are asked to think about how their parenting is the same or different from the scenarios presented in videos. |
| ^a^ Indicates plans for future development. ^b^Other aspects of social support (e.g., normative influence, social facilitation, cooperation) may be developed in a future third phase of development based on results of feasibility trial. Current data from the parent survey indicates this is not a priority. | | |
