# Supplementary material for: Designing a Mobile App to Enhance Parenting Skills of Latinx Parents: A Community-Based Participatory Approach
Source: JMIR Form Res. 2020 Jan 24;4(1):e12618. doi: 10.2196/12618 (PMC7007588; doi:10.2196/12618)
Supplement: Multimedia Appendix 2 [file formative_v4i1e12618_app2.docx]

Multimedia Appendix 2. Examples of Interview Questions

| Parent Interview 1 | |
| --- | --- |
| Technology | 1. Tell me about the technologies that you use.    1. What about the technologies that you don’t use?    2. What wearable technologies (like a fitbit) have you tried?    3. What apps do you use? 2. What technologies do you use when you feel stress?    1. What technologies to you not use when you feel stress? |
| Use of Technology for Parenting | 1. What technologies or online resources do you use for parenting? 2. What do you find challenging about using technology? 3. What kinds of technology or online resources would you like to see available for parents? 4. Tell me about how technology or online resources help you as a parent.   a. How would that resource help you with parenting stress?   1. How does using technology or online resources affect the relationship you have with your children?   a. How does technology complicate being a parent? |
| Preferences | 1. Think about the features in each example set of an app. Which would be the most important and least important characteristics to you? |
| Feedback on Mockup | 1. First, look at this page, and tell me what you think of it. What stands out to you? For example, who is it for, what is its purpose, and what is it for? 2. Now I’m going to ask you to try doing some specific tasks.    1. If you had a hard day at work and wanted to relax for two minutes before helping your child do homework, what part of the app might be helpful?    2. If you wanted to find information about how to discipline your teenager when they are out late, what would you do?    3. If you wanted to see how well you have been doing on goals you made this week, what would you do?    4. If you were wearing a wristband that measured your stress, where could you see information about your stress levels? 3. Could you see yourself wearing a wearable device like this? Why or why not? 4. How might getting feedback on your breathing affect you |
| Parent Interview 2 | |
| Feedback on Prototype |  |
| Feedback on Wearable |  |
