# Supplementary figures and images for: Designing a Mobile App to Enhance Parenting Skills of Latinx Parents: A Community-Based Participatory Approach
Source: JMIR Form Res. 2020 Jan 24;4(1):e12618. doi: 10.2196/12618 (PMC7007588; doi:10.2196/12618)

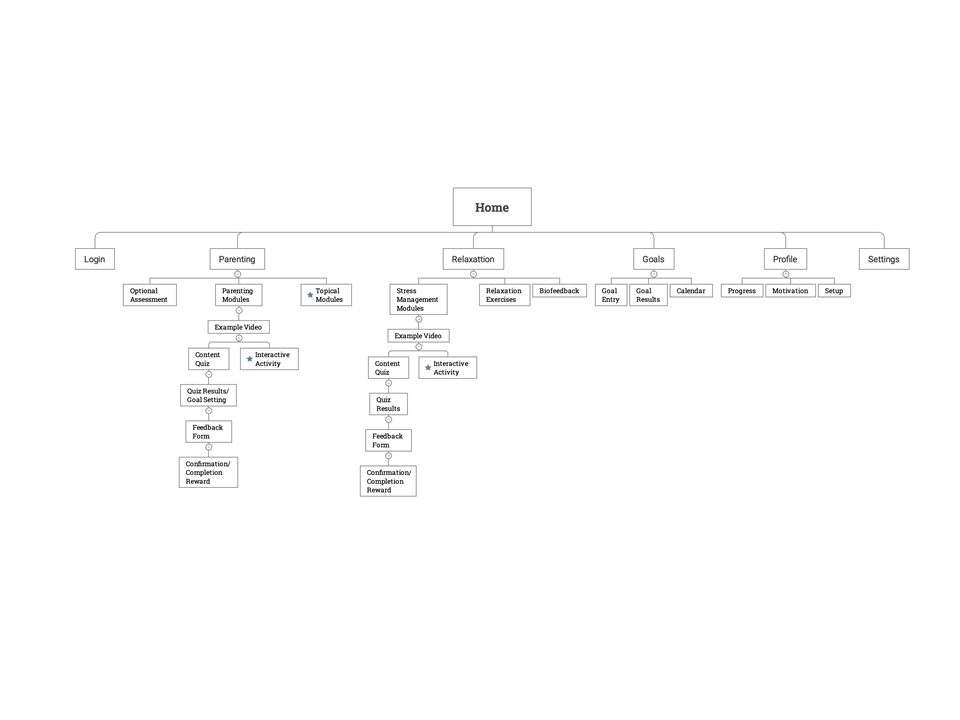

Supplement: Multimedia Appendix 3 [file formative_v4i1e12618_app3.png]
